# CAMIO: a transgenic CRISPR pipeline to create diverse targeted genome deletions in *Drosophila*

**DOI:** 10.1093/nar/gkaa177

**Published:** 2020-03-18

**Authors:** Hui-Min Chen, Jorge Garcia Marques, Ken Sugino, Dingjun Wei, Rosa Linda Miyares, Tzumin Lee

**Affiliations:** Howard Hughes Medical Institute, Janelia Research Campus, 19700 Helix Drive, Ashburn, VA 20147, USA

## Abstract

The genome is the blueprint for an organism. Interrogating the genome, especially locating critical *cis*-regulatory elements, requires deletion analysis. This is conventionally performed using synthetic constructs, making it cumbersome and non-physiological. Thus, we created Cas9-mediated Arrayed Mutagenesis of Individual Offspring (CAMIO) to achieve comprehensive analysis of a targeted region of native DNA. CAMIO utilizes CRISPR that is spatially restricted to generate independent deletions in the intact *Drosophila* genome. Controlled by recombination, a single guide RNA is stochastically chosen from a set targeting a specific DNA region. Combining two sets increases variability, leading to either indels at 1–2 target sites or inter-target deletions. Cas9 restriction to male germ cells elicits autonomous double-strand-break repair, consequently creating offspring with diverse mutations. Thus, from a single population cross, we can obtain a deletion matrix covering a large expanse of DNA at both coarse and fine resolution. We demonstrate the ease and power of CAMIO by mapping 5′UTR sequences crucial for *chinmo's* post-transcriptional regulation.

## INTRODUCTION

Understanding how complex biology unfolds, advances, and evolves from genomic sequences requires development of precise and efficient tools to interrogate the genome. While spontaneous or induced mutations once served as the basis of our understanding about many biological processes, we now exploit sophisticated methods to directly manipulate intact genes for mechanistic studies.

Unlike in coding sequences, no overarching rules exist to help us identify functional elements in noncoding regions. There are bioinformatics resources for predicting *cis*-regulatory elements (CREs) (1,2). Also, locations of CREs can be inferred from chromatin state (3–6). However, such methods are restricted to canonical genomic signatures. Moreover, these putative CREs need to be assessed molecularly to determine whether they are functional. Originally, molecular biologists relied heavily on artificially constructed assays to assess the function of DNA fragments, such as promoter bashing (7,8) and enhancer screening (9–11). More recently, thanks to the advancement in DNA synthesis and sequencing capacity, high-throughput platforms have been designed to survey CREs on a genome-wide scale (12–15). However, these approaches are intrinsically artificial and indirect, and most unsatisfactorily preclude studying regulatory elements in their native environment.

To uncover functions of noncoding sequences, we need ways to efficiently manipulate them *in-situ*. The invention of tailor-made sequence-specific DNA nucleases, particularly CRISPR (clustered regularly interspaced short palindromic repeats) greatly facilitate such efforts. CRISPR was first exploited for targeted mutagenesis (16) and then swiftly adopted to edit genomes of diverse organisms (17–22). CRISPR’s popularity lies in its simplicity: a Cas9 nuclease plus an easily synthesized guide RNA (gRNA) induces a double-strand break (DSB) targeted by DNA–RNA base pairing (16,18). Cells repair the DSBs through nonhomologous end joining (NHEJ) (23), leading to insertion or deletion (indel). The fact that CRISPR can produce targeted DNA modifications with near unlimited specificity has promised its rapid expansion throughout the biological and biomedical fields (24).

Large-scale screening for regulatory elements with CRISPR includes targeted disruption (25–27), CRISPRi repression (28), or CRISPRa activation (29) with vast gRNA libraries in cell culture. Targeted disruption suffers from insufficient perturbation of genome with small indels, and for CRISPRi and CRISPRa, the spreading epigenetic effect from the inhibition or activation enzyme domains also makes CRE prediction less precise. To overcome these limitations, tiling deletion screenings with a paired gRNA library have been performed to examine large sections of genomes with defined overlapping deletions (30,31). Larger deletions have a better chance to disrupt regulatory elements in their native context. These large-scale CRISPR regulatory screenings with huge gRNA libraries have great potential to unbiasedly interrogate long stretches of genome in a high-throughput manner. However, the cell culture-based platforms undoubtfully limit the types of biology one can investigate. For most spatially, temporally, or tissue-specifically defined biological questions, we believe deletion analyses should be equipped with a pipeline that systematically produces mutant organisms with diverse targeted mutations.

We envision such a pipeline in *Drosophila melanogaster*. The fruit fly has been a powerful model organism for decoding the genome to understand complex biology (32). We desire a fully *Drosophila* transgenic system that is easily scalable and automatically produces a variety of deletions in a targeted region. To build a CRISPR pipeline with germline transmission, we need Cas9 expressed in the reproductive system. Traditional *Drosophila* germline drivers, such as *nosP* or *vasaP*, drive expression in germline stem cells, thus limiting the number of possible mutations per founding parent (33). However, *bamP*, a germ cell specific driver that activates expression specifically in primordial germ cells (34,35) would ensure distinct CRISPR events in each germ cell. This would therefore provide every progeny with a *de novo* mutation.

Here, we describe a new technology for comprehensive deletion analysis called CAMIO (Cas9-mediated Arrayed Mutagenesis of Individual Offspring). We built a CRISPR-based mutagenesis pipeline in *Drosophila* male germ cells, to achieve massive production of independent indels in targeted loci with germline transmission (34,36). Further, we created a transgenic system for simultaneously targeting multiple sites with an array of guide RNAs. This way, we can readily generate a huge collection of organisms harboring either diverse, small, localized indels or large, defined deletions (inter-target deletions). This enables efficient deletion analysis of sizable genomic regions *in vivo*. We include here an example that demonstrates the power of CAMIO. In 2006, our lab discovered that the expression of temporal protein, Chinmo, was regulated via its 5′ untranslated region (UTR) (37). Using CAMIO, we were able to rapidly ascribe a critical aspect of this temporal control to a 154-bp sequence of the 2 kb UTR.

## MATERIALS AND METHODS

### Fly strains

We used the following fly strains in this work: (i) *bamP(898)-Cas9* in *attP2*; (ii) *U6:3-gRNA-e* (21); (iii) *Df(3R)ED10838/TM2* (BDSC #9485); (iv) *dU6-3-gRNA-shi* in *attP40*; (v) *UAS-shibire* in *attP2*; (vi) *GMR-Gal4*; (vii) *nos-phiC31-nls #12*; (viii) *10XUAS-mCD8::GFP* in *attP40* (38); (ix) *act5C-Gal4/TM6B*; (x) *pCis-{4gRNAs_mCD8}* P element insertion line #3, #10 on II and #9, #12 on III; (xi) *13XLexAop2-5*′ *UTR-smGFP-OLLAS* in *VK00027*, *13XLexAop2 –smGFP-cMyc-3*′ *UTR* in *attP40* and *13XLexAop2-5*′ *UTR-smGFP-V5-3*′ *UTR* in *su(Hw)attP5*; (xii) *41A10-KD*; (xiii) *DpnEE-KO-LexAp65*; (xiv) *pCis-{4gRNAs_chinmo Exon1}* P element insertion line #23, #25 on II and #4, #5 on III; *pCis-{6gRNAs_chinmo Exon2}* #7, #11 on II, and #14 on III; *pCis-{4gRNAs_chinmo Exon3}* #5 on II, and #2, #3 on III; (xv) *FRT40A,UAS–mCD8::GFP,UAS–Cd2-Mir/CyO,Y* (39); (xvi) *OK107-Gal4*; (xvii) *UAS-Cas9* (21); (xviii) *dU6_g2+3* in *VK00027*; (xix) *Dpn-Cas9* (40); (xx) *act5C-Cas9* (21); (xxi) *13XLexAop2-53UTR-smGFP-V5-d*, *13XLexAop2-53UTR-smGFP-V5-bD* and *13XLexAop2-53UTR-smGFP-V5-D23* in *attP40*. 22. *nosP-Cas9* (BDSC #54591) and *vasaP-Cas9* (BDSC #51324).

### Molecular biology

To create *bamP(898)-Cas9*, the full *bam* promoter (-898) (34) was ordered from gBlocks, IDT and Cas9 was also flanked by *bam* 3′ UTR. To create UAS-shibire, codon-optimized *shibire* coding sequence carrying the gRNA-shi target site was ordered from GeneArt gene synthesis, and then cloned into pJFRC28 (41). The UAS-shibire transgene was designed to carry multiple silent mutations around the gRNA target site so that only the transgene and not the endogenous gene is targeted. To generate dU6-3-gRNAs, we replaced 10XUAS-IVS-GFP-p10 of pJFRC28 with dU6-3 promoter and gRNA scaffold fragment from pTL2 (36). For dU6-3-gRNA-shi, GTATGGGGTATCAAGCCGAT was selected as the spacer. To create dU6_g2+3, we first generated dU6-3-g2_2 and dU6-3-g2_3 separately and cloned dU6-3-g2_2 into the backbone of dU6-3-g2_3.

To create conditional U6-gRNA set construct, pCis-{4gRNAs_mCD8}, a U6 promoter-AttB fragment was synthetized by PCR amplification from pCFD3 (21) and cloned into pCaST-elav-VP16AD, which contained the p-Element inverted repeats (Addgene, #15308). Then, we inserted a DNA fragment (Genscript) containing 4 different gRNAs targeting the mCD8 protein tag. These gRNAs were selected based on their ON and OFF target scores (Benchling) for potent mutating strength and minimal off-target mutagenesis (42,43). An off-target score of 50 or higher is considered to be for a gRNA with very low chance of an off-target event. The gRNA with the lowest off-target score of 43.7 was chosen out of necessity, while the rest of them are all above 46. Each of these gRNAs was preceded by an AttP site and a HammerHead ribozyme (44). Finally, a 3Xp3-RFP-polyA(α-tubulin) fragment was synthetized by PCR amplification, using pure genomic DNA from a fly line in which this cassette was used as a marker (Bloomington, #54590). Then, this fragment was inserted upstream of this gRNA region. In the final construct, the AttB and AttP sites were separated by a 3.7 kb region containing an ampicillin resistance gene, an origin of replication in bacteria and the 3Xp3-RFP-polyA marker.

pCis-{gRNAs_chinmo-Exon1/Exon2/Exon3}: following the same design described above, a DNA fragment was synthetized (Genscript), which contained four gRNAs (six for Exon2) either targeting the corresponding exon or the exon-intron junction. This fragment was then inserted into pCis-{4gRNAs_mCD8}, thus removing the previous gRNAs cassette.

The *Chinmo* UTRs were amplified from Drosophila genomic DNA. smGFP (45) fused to V5, cmyc or ollas were amplified from previously existing plasmids. Standard molecular biology techniques were used to clone the smGFP fusions containing one or two *Chinmo* UTRs into 13XLexAop2 (pJFRC15, (38)). If the construct ended with *Chinmo* 3′UTR, the SV40 signal was removed from the vector backbone. *13XLexAop2*-*5*′ *UTR-GFP-3*′ *UTR* was further modified to create d, bD and D23 reporters containing various deletions in the 5′ UTR.

### *Drosophila* genetics

#### *ebony* and UAS-shibire mutagenesis

Female or male founders (*bamP-Cas9*, *nosP-Cas9* or *vasaP-Cas9* with *U6:3-gRNA-e*) were mated to *Df(3R)ED10838/TM6B*, and chromosomes over *Df(3R)ED10838* were scored for ebony loss-of-function phenotype. Male *UAS-shibire* mutagenesis founders were crossed with *GMR-Gal4*, and the wildtype-eyed 919 progeny were sacrificed for next generation sequencing (NGS).

#### CAMIO on UAS-mCD8::GFP

Male founders (*UAS-mCD8::GFP*, *bamP(898)-Cas9*, *nos-phiC31* and one or two copies of *pCis-{4gRNAs_mCD8}* were mated with *act5C-Gal4* females for scoring of loss of green fluorescence in the progeny. For one copy of *pCis-{4gRNAs_mCD8}*, we screened 807 progeny from 20 founder males. Thirty loss-of-GFP progeny from two founders were further subject to sequence analysis. For two copies of *pCis-{4gRNAs_mCD8}*, we screened 570 progeny from 16 founders. One hundred and ten loss-of-GFP progeny were analyzed and grouped into three deletion categories.

#### CAMIO on *chinmo* 5′ UTR

After mating with females carrying a second chromosome balancer for stock keeping, 1200 male progeny from 4 CAMIO on *chinmo* 5′ UTR gRNA set combinations were sacrificed for genomic study. We designed primer sets that produce amplicons covering exon 1, 2, 3 and exons 1+2. To save on the cost, we deliberately mixed amplicons from different exons and barcoded them together so that with 384 (96 × 4) Nextera barcodes, we managed to sequence 984 (300 × 3 + 84) CAMIO progeny. Males from combination 1 were intentionally numbered 1–300, and their genomic amplicons were carefully matched and mixed with counterparts from combinations 2 and 3. Finally, 300 DNA mixtures plus 84 amplicons from combination 4 were tagmented (Nextera XT DNA Library Prep Kit, illumina) and barcoded (Nextera XT Index Kit v2) for NGS.

### Immunohistochemistry and confocal imaging

Fly brains at indicated larval, pupal, and adult stages were dissected, fixed and immunostained as described previously (37,39). The following primary antibodies were used in this study: chicken GFP polyclonal antibody (1:500, Invitrogen, A10262); rat anti-Deadpan (1:100, abcam, ab195173); mouse 1D4 anti-Fasciclin II (1:50, DSHB); rabbit polyclonal anti-Trio (1:1000) (46); rat anti-Chinmo (1:500, a gift from the Sokol lab) (47). Secondary antibodies from Molecular Probes were all used in a 1:200 dilution. The immunofluorescent signals were collected using Zeiss LSM 880 confocal microscope and processed using Fiji and Adobe Illustrator.

### Bioinformatics

Sequence reads (FASTQ data) were first processed with cutadapt [https://github.com/marcelm/cutadapt] to remove adapter sequences with options: –overlap = 7 – minimum-length = 30 -a ‘CTGTCTCTTATACACATCTCTGAGCGGGCTGGCAAGGCAGACCG’. Then they were mapped to the genomic sequence corresponding to the *chinmo* region using Bowtie2 (48) with the following options: –local –score-min G,20,0 -D 20 -R 3 -L 20 -i S,1,0.50 –no-unal. Resulting SAM files were parsed with Pysam [https://github.com/pysam-developers/pysam] to extract deletion/insertion information from the Cigar strings. When the cigar string contained ‘D’ or ‘N’, we extracted mapped sequences as deletions and designated them as type D. When the cigar string contained ‘I’, we extracted them as insertions and designated them as type I. While parsing the SAM file, soft clipped reads (reads partially mapped to the genome) were detected and clipped (unmapped) portions were set aside in a FASTA file. The sequences in this FASTA file were then re-mapped using bowtie2 to the genomic sequence encompassing the *chinmo* gene. Then, mapped portions of the first mapping and that of the second mapping (when the second one existed) were merged to form a deletion event which we denoted as type L (large gap). This type of gap often contained inserted sequences in the middle. We discarded any events with less than 10 reads.

## RESULTS

### Independent targeted mutagenesis in individual male germ cells

To efficiently and comprehensively perform deletion analysis of a genomic region, we envision a mutagenesis pipeline where multiple mutations are produced from a single founder animal. This could be best achieved by inducing mutagenesis in individual germ cell rather than the commonly used germline stem cells (GSC). The gene, *bam*, is restricted to germ cells in both the male and female germline. Moreover, we have previously shown in the female fly that the *bam* promoter can effectively and specifically drive flippase induction in the germ cells, and not in GSCs (36). Here we explored whether we could use *bam* in the male to drive independent mutagenesis in individual sperm.

**Figure 1.**
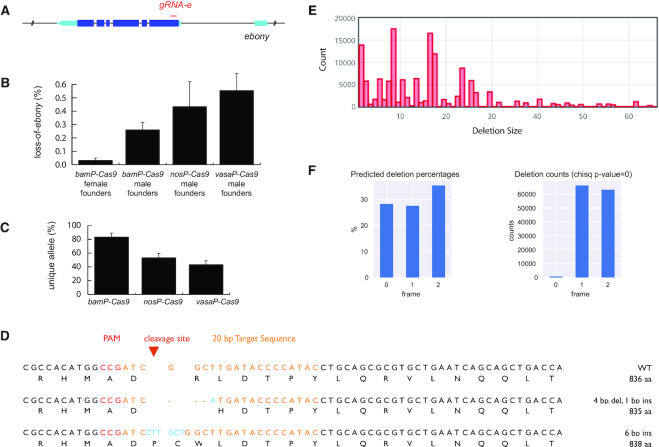
*bamP-Cas9* induces efficient CRISPR targeted mutagenesis in male germ cells. (**A**) Ebony transcript shown with UTRs in turquoise. U6 drives guide RNA (*gRNA-e*), which targets 5′ end of *ebony* coding sequence. (**B**) Percent of progeny with ebony loss-of-function (LOF) mutations from female or male founders. Mean ± SD (*n* = 10). (**C**) Percent of progeny with unique ebony alleles. Mean ± SD (*n* = 3). (**D**) Sequences of two in-frame *shibire* mutations. (**E**) Deletion profile of *UAS-shibire* transgene following CRISPR targeting collected from 919 phenotypically wildtype progeny. NGS data was analyzed and presented by Cas-analyzer, www.rgenome.net (64). (**F**) Left, percentage of predicted indels (calculated by FORECasT (57) using *gRNA-shi* and *UAS-shibire* sequences) grouped into three reading frames, 0 represents the in-frame indels. (Right) Actual reading frame percentages from phenotypically wildtype progeny, calculated from mapping results (obtained with CAS-analyzer). Chi-square test assuming equal distribution was used to assess the significance. The result is below machine precision and thus set to zero.

To this end, we made *bamP(898)-Cas9* and tested its ability to mutate gRNA-targeted sites in male as well as female germline. For proof of principle, we chose the *ebony* gene for targeted mutagenesis. Loss-of-*ebony* mutations are easy to detect and there was a readily available transgenic gRNA targeting *ebony* (Figure 1A) (21). We determined the mutagenesis efficiency in male versus female founders (Figure 1B). Surprisingly, over 25% of male progeny as opposed to only ∼3% of female ones carried loss-of-function *ebony* mutations. This result demonstrates that the male germline is particularly susceptible to *bamP-Cas9*-mediated genome editing.

**Figure 2.**
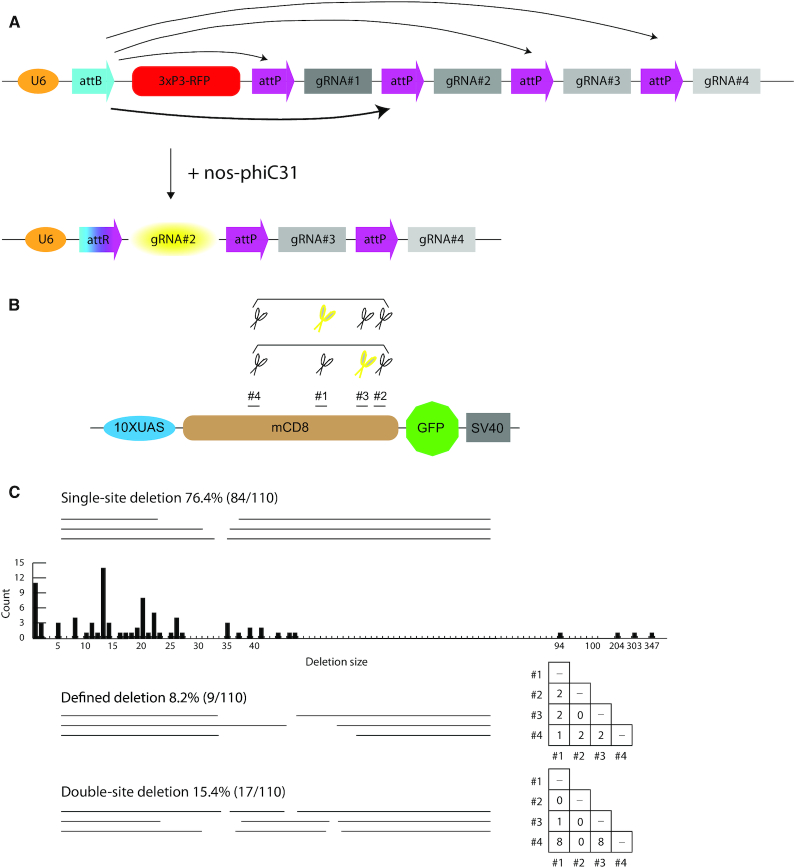
CAMIO produces diverse arrayed mutations around selected gRNA target sites. (**A**) Schematic of a conditional U6 gRNA transgene: *pCis-{4gRNAs_mCD8}*. The U6 promoter is separated from the gRNAs by a large fragment containing a 3xP3-RFP marker. With phiC31 recombination, the attachment site, attB can recombine stochastically with any of the four attP sites (black arrows), creating attR. The phiC31 recombinase is expressed in GSCs, controlled by *nos*. After recombination, a single gRNA is under the control of the U6 promoter (yellow oval). (**B**) Four gRNA target sites along the mCD8 coding sequence were selected for pCis-{4gRNAs_mCD8} to disrupt mCD8::GFP expression. Simple or combined multiplexing mutagenesis can be achieved by incorporating one or more *pCis-{4gRNAs_mCD8}* transgenes. Here, we show two transgenes, each represented by four pair of scissors. Stochastically chosen gRNAs in each set are further marked in yellow. (**C**) Three categories of deletions arose from applying two copies of *pCis-{4gRNAs_mCD8}*: single-site deletion, defined deletion and double-site deletion. Deletion size and count are depicted for single-site deletions. Right, use matrix of gRNA choices, deduced from sequencing GFP negative progeny.

To confirm that *bamP-Cas9* indeed creates a mutagenesis pipeline—producing an assortment of different alleles with a single founder—we compared *bamP-Cas9* driven mutagenesis with that of the traditional GSC mutagenesis via *nosP-Cas9* or *vasaP-Cas9*. We used the same gRNA targeting ebony and compared both mutation efficiency (Figure 2B) and the ability to produce diverse alleles (Figure 2C). As *nosP* and *vasaP* give Cas9 an earlier onset of expression in GSCs, we expect to see multiplication of mutational events occurring in the GSCs. Not surprisingly, *nosP-Cas9* and *vasaP-Cas9* male founders produced a higher percentage of loss-of-*ebony* progeny (43.5% and 55.5%), but their mutation rates also varied more individually, possibly due to limited GSCs each founder has (Figure 2B). To assess mutational variety, we sequenced 30 loss-of-*ebony* progeny from each condition (10 progeny each from three different male founders), and found that with *bamP*, an average of 83% of progeny contained unique alleles, compared to ∼50% when Cas9 is expressed in the GSCs (Figure 2C). This lower number is due to obvious clonal expansion from both *nosP-Cas9* and *vasaP-Cas9* male founders (Supplementary Figure S1). Therefore, we believe *bamP-Cas9* is an optimal mutagenesis pipeline design for diverse mutations.

A mutagenesis pipeline could be useful to produce novel alleles of a protein of interest. To explore this idea, we mutagenized a well characterized gene, *shibire. shibire* encodes *Drosophila* Dynamin, a motor protein crucial for synaptic vesicle endocytosis (49). *shi^ts1^*, a dominant temperature sensitive allele containing a missense mutation (G268D), is widely used to temporarily shut off neuronal activity in behavioral assays (50). We therefore decided to target a *UAS-shibire* transgene in the same region mutated in the *shi^ts1^* allele. The target area of the transgene was altered with silent mutations to maintain the translated amino acid sequence but create a unique DNA sequence for gRNA targeting. In this manner, only the transgene and not the endogenous *shibire* is targeted. We would expect new dominant negative (DN) *shibire* alleles created by CRISPR to be caused by in-frame mutations. A frameshift would lead to a premature stop codon, and the resultant small truncated protein would be likely non-functional. By contrast, in-frame mutations would create essentially full-size proteins. Furthermore, loss of critical amino acids could disrupt key catalytic functions while preserving the protein's ability to polymerize, thus creating a DN allele.

We expected overexpressing DN *shibire* mutations could disrupt the development of the delicate *Drosophila* compound eyes. Therefore, male founders (*bamP(898)-Cas9*, *dU6*-*3-gRNA-shi*, *UAS-shibire*) were mated with a Gal4 line that drives expression in the eye (*GMR-Gal4*). The resulting progeny were raised at 29°C to screen for rough eye phenotypes and thus new DN *UAS-shibire* alleles. In our small initial selection of rough eye progeny to sequence, we collected two in-frame mutations of *shibire* as predicted (Figure 1D). Unfortunately, we also collected many progeny with no mutations in the *UAS-shibire* transgene. We realized that high *GMR-Gal4* expression can lead to the rough eye phenotype when *UAS-shibire* is not mutated. As the strength of *GMR-Gal4*—and therefore rough eye phenotype—was highly variable, it was impossible to discern which progeny had rough eyes due to mutations in *UAS-shibire* and which were due to *GMR-Gal4* overexpression.

Because of the unexpected flaw in the experimental design, we were unable to accurately assess which mutations in the transgene are putative DN mutations. We reasoned, however that DN alleles would be absent from offspring with normal eyes. If, as we expect, loss of amino acids in the targeted region are most likely to cause DN mutations, such indels should be underrepresented in the phenotypically normal progeny. We therefore surveyed phenotypically wildtype offspring. We pooled ∼1000 phenotypically normal progeny collected from 20 male founders for amplicon analysis with next generation sequencing (NGS). We obtained a large collection of diverse indels, with the majority of deletions smaller than 30 bp (Figure 1E). Notably, there is a clear under-representation of in-frame mutations (Figure 1F). This suggests that loss of one or more amino acids in the targeted region does lead to novel alleles which result in the rough eye phenotype. These experimental results demonstrate both that a mutagenesis pipeline can serve to produce novel alleles of a gene of interest and that an appropriate screening platform is crucial to identify and recover said alleles.

Taken together, our data demonstrate that *bamP(898)* effectively restricts Cas9-mediated mutagenesis to germ cells. There is no evidence that clonal expansion contributes to the exceptionally high mutation efficiency in male founders. Therefore, transgenic CRISPR, induced by *bamP*, can effectively serve as a pipeline for mass production of targeted mutations.

### CAMIO: Cas9-mediated arrayed mutagenesis of individual offspring

Despite independent mutagenesis in each germ cell, using a single gRNA limits the offspring variation, as all indels are anchored around the same Cas9 cut site. To expand the diversity of deletions one can recover from a single population cross, we next explored the possibility of multiplexing gRNA-targeted mutagenesis. Our vision for multiplexing gRNAs is to have a collection of gRNAs from which one is stochastically selected, rather than simultaneously expressing multiple gRNAs. Incorporating this multiplexed design into the male germline in combination with *bamP(898)*-Cas9 would enable both stochastically chosen gRNAs and offspring with independent mutations. Supplying one gRNA at a time prevents contamination of rare deletions by much more frequent second-site mutations. This way, discrete clusters of simple deletions can be recovered from a single population cross. Therefore, we can tile a sizable DNA region with diverse small deletions with a repertoire of evenly spaced gRNAs.

To examine the feasibility of our multiplexing design, we targeted a *UAS-mCD8::GFP* transgene with four independent gRNAs. To stochastically activate only one out of the four gRNAs, we made a conditional U6-gRNA(x4) transgene that is dependent on PhiC31-mediated recombination (Figure 2A). Using the *nos* promoter, we control the induction of the phiC31 recombinase in GSCs. Thus, in each of the 12–24 GSCs per male fly founder (33), the transgene is irreversibly recombined to express a single gRNA. Recombination occurs between a single attB site downstream of the U6 promoter and a choice of attP sites upstream of each gRNA; once reconstituted, the ubiquitous U6 promoter drives expression of only one of the gRNAs. The intra-chromosomal recombination excises an intervening 3xP3-RFP. Given the rather small size of each gRNA as compared to the large 3xP3-RFP, the differences in length between the attB site and the choice of any one attP site is therefore relatively trivial. Based on a previous, similar construct for multicolor imaging (51), we expect that each gRNA should be expressed at comparable frequencies. For brevity, we name the conditional U6-gRNA transgene pCis, and then, in braces, add the number of gRNAs and the name of the targeted DNA. For example, to target the *UAS-mCD8::GFP* transgene, we created pCis-{4gRNAs_mCD8}. Also, when we describe the individual gRNAs, we number them in sequence from 5′ to 3′.

For the multiplexed targeted mutagenesis of mCD8::GFP, we established male founders carrying *UAS-mCD8::GFP*, *bamP(898)-Cas9*, *nos-phiC31*, and *pCis-{4gRNAs_mCD8}*, and crossed them to *act5C-Gal4* females for easy scoring of GFP fluorescence in the progeny. Overall, ∼35% of the progeny lost GFP expression. We collected 30 GFP-negative offspring from two founder males. Sequencing the mCD8-coding region revealed that each GFP-negative offspring carried an indel corresponding to a single gRNA (Supplementary Figure S2). Encouragingly, we recovered various deletions resulting from activation of each of the four gRNAs. However, the frequency of mutations at each target site varied. Both founders yielded many more deletions around the gRNA#1/#4 targets than the gRNA#2/#3 targets, possibly reflecting their different on-target potencies.

We found that the majority (83.3%) of deletions removed 20 or fewer bp and that the largest one eliminated 85 bp. To tile a sizable DNA region with such small deletions would require many gRNAs bombarding the region of interest at a density of around one gRNA per 100 bp. Alternatively, we should be able to create larger deletions spanning two Cas9 cuts elicited by two gRNAs acting at a distance. To explore co-employment of two gRNAs, we provided two copies of *pCis-{4gRNAs_mCD8}* for multiplexed dual mutagenesis of *UAS-mCD8::GFP* (Figure 2B). We obtained a comparable loss-of-GFP mutation rate at ∼35% despite co-expressing either identical or distinct U6-gRNAs. This phenomenon implies that Cas9 activity (either the level or duration) limits the efficiency of gRNA-directed mutagenesis in germ cells. Nonetheless, we could recover various diverse mutations from the dual gRNA-derived GFP-negative progeny, including many single-site deletions (76.4%) and quite a few double-site deletions (two target sites with independent indels; 15.4%) as well as some large deletions spanning two gRNA target sites (inter-target deletions; 8.2%) (Figure 2C). Notably, the single-site deletions greatly outnumbered those involving two sites. This outcome is favored in large-scale deletion analysis, as it increases the chance of recovering deletions without second-site contamination.

The above results demonstrate that using dual gRNA sets enables us to tile a region of interest not only with indels, but also with defined deletions. Random selection of a single gRNA from each of the two identical sets which contain four gRNAs will yield six possible defined deletions. Encouragingly, from a collection of only nine inter-target deletions, we recovered five of the six anticipated defined deletions. Nevertheless, physical hindrance may prevent two Cas9 complexes from acting simultaneously on very close gRNA targets. This may explain why we failed to recover the smallest defined deletion of 37 bp between the Cas9 cut sites of gRNA#2 and #3 targets. These results suggest that inter-target deletions utilizing two gRNAs can support rapid systematic DNA deletion analysis.

In sum, we established an effective strategy to express various permutations of two gRNAs in male GSCs. In combination with restricting Cas9 to male germ cells, we built a germline pipeline for multiplex targeted mutagenesis. We dub this genetic system CAMIO (Cas9-mediated arrayed mutagenesis of individual offspring), which can derive from a single population cross a matrix of variable deletions. This strategy enables deletion analysis of a substantial DNA region with both coarse (inter-target deletions) and fine (a variety of single- or double-site deletions) resolution. Below, we prove the power of CAMIO in structure–functional analysis of a 2.2 kb-long genomic fragment.

### Structure–functional analysis of *chinmo* 5′UTR

The Chinmo BTB-zinc finger nuclear protein is dynamically expressed in intricate spatiotemporal patterns in the developing *Drosophila* central nervous system. Such dynamic Chinmo expression governs various aspects of temporally patterned neurogenesis, including age-dependent neural stem cell proliferation (52,53) and birth order-dependent neuronal cell fate (37,54,55). Notably, *chinmo* transcripts exist much more broadly than Chinmo proteins, indicating involvement of negative post-transcriptional regulation (37,53). Consistent with this notion, *chinmo* transcripts have long UTRs, including a 2.2 kb 5′UTR and an 8.5 kb 3′UTR (47). Our lab previously described a role for the *chinmo* 5′UTR in graded translation of Chinmo protein in adult MB neurons (37). A subsequent paper described control of larval Chinmo expression via miRNA *let-7* binding sites on the 3′UTR (47), which was not fully annotated in our original assessment (37). We therefore set about to first validate the role of the 5′ UTR in Chinmo translation regulation (37), followed by structure function analysis of the UTR using CAMIO.

We started by making GFP reporter transgenes carrying *chinmo* 5′ and/or 3′ UTR(s). For a functional readout, we utilized the development of the *Drosophila* mushroom body (MB), which involves an orderly production of γ, α’β’, and αβ neurons. We first determined the roles of the 5′ vs. 3′UTR in downregulation of Chinmo expression along MB neurogenesis (37), by examining the change in expression of GFP reporter transgenes (containing either or both UTRs) from early to late larval stages (Supplementary Figure S3). Notably, presence of the *chinmo* 5′UTR drastically suppressed the reporter expression. Interestingly, only in the absence of the 3′UTR did we detect an enhanced 5′UTR-dependent suppression at the late larval stage. These phenomena ascribe the *chinmo* downregulatory mechanism(s) to the 5′UTR, and unexpectedly revealed some upregulation by the 3′UTR. This upregulation could potentially be a transgene-specific artifact, arguing for the importance of performing assessments in the native environment. We thus turned to CAMIO to carry out structure-functional analysis of the native *chinmo* 5′UTR.

**Figure 3.**
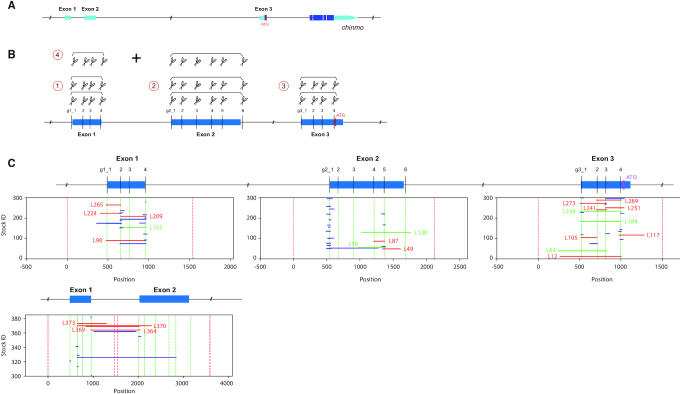
Applying CAMIO on *chinmo* 5′ UTR. (**A**) An illustration of the *chinmo* gene, UTRs are depicted in turquoise. (**B**) We selected four target sites on *chinmo* exon 1 (455 bp), six target sites on exon 2 (1061 bps), and four target sites on exon 3 (639 bp). Each exon was dissected by CAMIO with a pair of gRNA sets, depicted as a set of scissors. Additionally, we combined gRNA sets from exon1 and exon2. (**C**) Representation of larger deletions (marked in blue) predicted by NGS of 984 offspring (300 each for individual exons and 84 for Ex1–Ex2). Predicted deletions over 100 bp were subject to Sanger sequencing for confirmation. Confirmed >100 bp deletions are marked in red and given an ID#. Selected homozygous viable deletions (marked in green) that cover larger proportions of each exons were selected for MB development studies.

*chinmo*’s 5′UTR is separated into three exons; the first two exons are neighboring and the distant third exon is separated from the second by 36 kb (Figure 3A). A separate gRNA set was designed to target each exon for CRISPR mutagenesis (Figure 3B). We provided two copies of the same set for induction of both indels and inter-target deletions within an exon. Additionally, we paired gRNA sets for exons 1 and 2 to create larger deletions that span the exon1/2 junction. Thus, we could delete various parts of *chinmo* 5′UTR in its endogenous locus using the automatic CAMIO mutagenesis pipeline.

Based on the previously observed deletion rate of around 35%, we collected 300 male offspring from each CAMIO genetic cross. We hoped to saturate each 5′UTR exon with ∼100 different deletions. In total, 1200 CAMIO males were harvested from the four different gRNA array combinations (exons 1, 2, 3 and 1+2). We mapped potential indels by sequencing indexed PCR products in a high-throughput manner. Briefly, we amplified the targeted exon (1, 2 or 3) from each of the selected progeny for each target, thus producing three sets of 300. We then combined an amplicon from each of the three sets for barcoding, as the introns are easily discernable from each other post sequencing. Separately, we amplified and barcoded 84 samples from exon 1+2. These numbers were chosen as the Nextera kit barcodes 384 samples.

**Figure 4.**
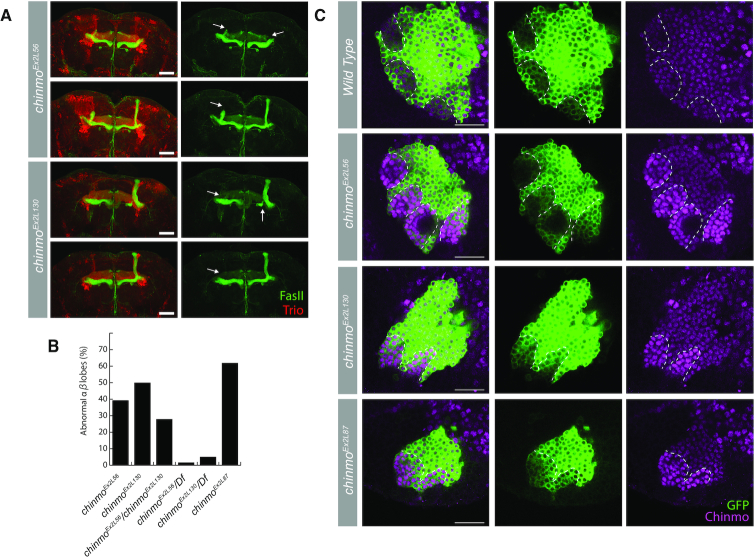
Overlapping deletions in 5′UTR alter Chinmo expression and MB morphology. (**A**) Stacked confocal images of adult MBs stained for FasII (green, αβ and γ lobes) and Trio (red, α’β’ and γ lobes). Missing or misshapen αβ lobes (arrows). Scale bars: 50 μm. (**B**) Percent of flies with abnormal αβ lobes. Homozygous and transheterozygous deletions have strong MB αβ lobe defects, whereas hemizygous deletions over a *chinmo* deficiency line (*Df*) are much less penetrant. (**C**) Single optical sections of MB lineages (*OK107-Gal4*, *UAS-mCD8::GFP*) immunostained for GFP and Chinmo at white pupa stage. Chinmo staining is elevated in newly derived MB neurons (weaker GFP signal, outlined by white dashed lines) in homozygous *chinmo^Ex2L56^*, *chinmo^Ex2L130^*, and *chinmo^Ex2L87^*. Green: GFP; Magenta: Chinmo. Scale bars: 20 μm.

We detected numerous indels around each gRNA target site (Supplementary Figure S4) and also recovered many inter-target deletions that together allow efficient coverage of the entire 5′UTR (Figure 3C). We made organisms homozygous for the large inter-target deletions and examined MB morphology. Markedly, we found similar aberrant MB morphology with two exon 2 inter-target deletions, *chinmo^Ex2L56^* and *chinmo^Ex2L130^* (Figure 4A). These deletions overlap by ∼300 bp, and prompted us to examine *chinmo^Ex2L87^*, a 154 bp deletion that lies within the overlapping region of *chinmo^Ex2L56^* and *chinmo^Ex2L130^*. Variable defects in the perpendicular projection of the bifurcated αβ axon lobes appeared at comparable frequencies (∼30–50%) in homozygous as well as transheterozygous brains. Further, the penetrance of this phenotype is sensitive to Chinmo dosage, as a *chinmo* deficiency line effectively suppressed the phenotype (Figure 4B). We and others have shown that Chinmo downregulation plays a role in promoting α′β′ to αβ MB neuron fate transition at the prepupal stage (37,56). Therefore, we assessed Chinmo levels, and observed aberrantly elevated Chinmo in young MB neurons around pupa formation in *chinmo^Ex2L56^*, *chinmo^Ex2L130^* and *chinmo^Ex2L87^* homozygous mutants (Figure 4C). This is consistent with the notion that these overlapping deletions have uncovered the essential region for this prepupal downregulation of Chinmo. In summary, a single round of CAMIO allowed us to identify a 154 bp locus in the 2.2 kb 5′UTR critical for proper MB development.

Meanwhile, notably absent were indels or large deletions involving the target sites g2_2 and g2_3. This area in exon 2 may carry essential sequences for Chinmo regulation that is critical for organism viability. Alternative explanations for the failure in recovering indels from that region include: a relatively shallow sequencing depth of the exon 2 region, our small sample size, unexpectedly low gRNA on-target strength for these gRNAs, or flawed design in the exon 2 gRNA set pCis-{6gRNAs_chinmo Exon2}. To address the last concern that bothered us most, we assessed the recombination efficiency for selection of each gRNA within the array to be downstream of the U6 promoter in *pCis-(6gRNAs_chinmo Exon2)* (Supplementary Figure S5). We created *pCis-(6gRNAs_chinmo Exon2)* progeny that underwent *nos-phiC31* mediated recombination. Importantly, we are examining only recombination of the gRNAs, rather than mutagenesis, as the experiment does not include Cas9. While g2_2 and g2_3 were not recruited as frequently as others, they each still emerged 6–7% of the time. A rate of 6–7% should be sufficient for us to recover some indels, as gRNA#1 was selected ∼15% of the time and produced multiple indels in our CAMIO experiment.

To examine whether this region of the 5′UTR is indeed critical, we exploited mosaic analysis to create somatic mutations in different tissues. Hence, a transgene, *dU6_g2+3*, was assembled to ubiquitously express both g2_2 and g2_3. We began with MB-specific CRISPR mutagenesis by inducing *UAS-Cas9* specifically in the MB lineage using a MB specific Gal4 (*OK107-Gal4*). We saw no temporal fate changes in the MB, the classic phenotype of *chinmo* misregulation. We next elicited CRISPR mutagenesis in all neural stem cells (neuroblast: NB) with NB-restricted Cas9 (*dpn-Cas9*) and *dU6_g2+3*. These animals were viable and showed no abnormal tumor-like NBs in larval or adult brains (typical of Chinmo overexpression) (53). These data do not support the presence of critical brain regulatory elements in the region targeted by g2_2 and g2_3. We strengthened this negative conclusion by directly removing various small-to-large fragments around g2_2 and g2_3 targets from the above *chinmo* UTR-containing GFP transgene. In developing MBs, we observed indistinguishable GFP expression profiles between wild-type and modified 5′UTRs (Supplementary Figure S6). In contrast to our negative findings in the brain, we found severe embryonic or early larval lethality when we induced early ubiquitous somatic mutations with *act5C-Cas9* and *dU6_g2+3*. This dominant lethality provides a direct explanation for our failure in recovering viable organisms carrying g2_2 or g2_3 induced indels. Together, these data suggest an essential role for *chinmo* 5′UTR outside of the brain.

In conclusion, a single round of CAMIO successfully led us to uncover two critical regions of the *chinmo* 5′ UTR. The first critical region lies around the g2_2 and g2_3 targets and carries essential sequences for organism viability, unrelated to Chinmo's functions in the brain. The second critical region of the chinmo 5′UTR was uncovered by the overlapping *chinmo^Ex2L56^*, *chinmo^Ex2L130^*, and *chinmo^Ex2L87^* deletions. We determined that this 154 bp region is essential to down-regulate Chinmo expression, ensuring proper MB development. This fruitful case-study exemplifies the power of CAMIO in systematic, unbiased deletion analysis of targeted genome regions.

## DISCUSSION

Two innovations synergistically enable CAMIO, a germline pipeline for arrayed CRISPR mutagenesis. First, the *bamP* promoter can specifically limit Cas9 endonuclease activity to individual male germ cells—thus individual offspring receive independent mutations. Second, the random-choice gRNA arrays provide extensive coverage for deletion analysis, with both small indels and large deletions. Hence, the combination of *bamP-Cas9* and gRNA arrays used in CAMIO enables *in vivo* targeted deletion analysis with both minimal molecular biology and minimal fly genetics work.

**Figure 5.**
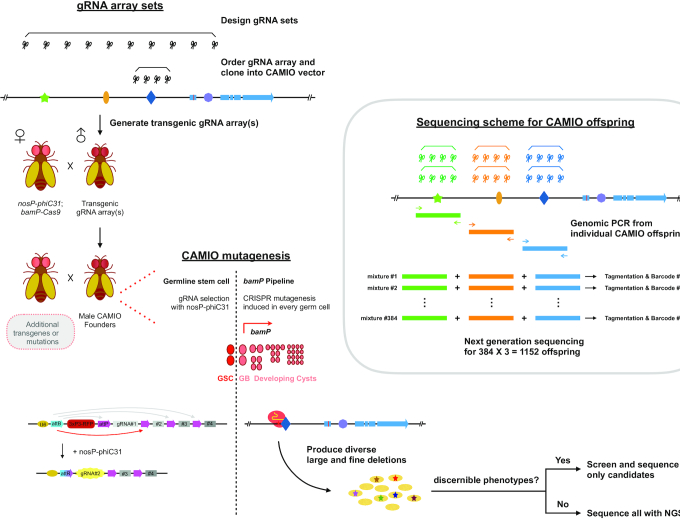
Implementing CAMIO deletion analysis. The first steps for CAMIO are designing gRNA arrays and creating transgenic flies. Within the arrays, gRNAs (scissors) can be widely spaced to investigate large DNA regions or narrowly spaced for fine dissection. First, select gRNAs with optimal ON and OFF target scores (e.g. benchling), these gRNAs can be assembled into an array by custom DNA synthesis (for details, see materials and methods). The DNA fragments is then cloned into U6/p-element CAMIO vector and sent for transgenesis. After acquiring transgenic gRNA arrays, these flies can be mated with *bamP-Cas9*; *nosP-phiC31* to create male CAMIO founders. Founder males can be crossed with females containing additional transgenes (e.g. Gal4/UAS or LexA/LexAop system) or mutations for phenotypic analysis or florescence screening. The subsequent CAMIO mutagenesis pipeline can readily produce offspring with targeted deletions. If a screening platform for discernible phenotypes is available, candidates can be quickly identified with Sanger sequencing. However, if a large number of CAMIO offspring needs to be molecularly characterized, a sequencing scheme with barcoding and NGS is provided for cost savings.

To apply CAMIO (Figure 5), one simply starts by designing a gRNA collection to dissect a genomic region of interest. gRNA selection can be assisted by in silico CRE prediction programs (1,2) or be unbiased, evenly spaced for an uncharacterized region. There are additional platforms to predict a gRNA’s mutating strength, off-target mutagenesis (42,43), and its likely indel profile (57). We used an online resource, Benchling, which helped us select optimal gRNAs with high ON and OFF target scores. Importantly, an off-target score of 50 or higher is for a gRNA unlikely to cause an off-target event. Given the current array design, it is possible to assemble ∼10 gRNAs into an array and also to employ multiple arrays for combinatorial mutagenesis. gRNA arrays can be ordered (custom DNA synthesis) and cloned into our CAMIO vector with p-element inverted repeats for commercial transgenic service. With the transgenic gRNA array sets at hand plus the *nosP-phiC31*, *bamP-Cas9* flies assembled by us, CAMIO can immediately produce diverse targeted mutants with large and fine deletions. Genotyping each mutant is essential to map the deletions. However, the number of CAMIO progeny to be sequenced can be limited to those that exhibit a phenotype of interest or those selected from a screening protocol. Indeed, CAMIO is easy to combine with preexisting tools such as the Gal4/UAS or LexA/LexAop binary systems for rapid fluorescence screening. If sequencing multiple mutants, sequencing costs and labor can be reduced by barcoding samples; if distinct DNA regions are targeted separately, the PCR amplicons can be combined when sample barcoding.

We were happily surprised to discover a much higher CRISPR mutagenesis rate in male compared to female germ cells using *bamP*. This sex difference was also observed in CRISPR-induced gene targeting in our effort to improve Golic+ (58). Currently, we do not know what leads to this phenomenon. *bamP* has a striking similar expression pattern in both the female and male germline: absence in GSCs and an early onset of expression during the four incomplete mitoses that produce the 16-cell germline cysts (35). One possibility is that the *bamP* activity may be higher in the male than the female germline. Another possibility has to do with the sex differences in meiotic recombination—meiotic recombination does not occur in male *Drosophila*. Perhaps reduced access to homologous chromosomes as templates for homology-mediated repair favors indels. In any case, this feature allows us to utilize *bamP* to build a high-efficiency pipeline for targeted CRISPR mutagenesis in male germ cells.

Conventional gRNA multiplexing provides all gRNAs at once as a cocktail, which expands indel diversity but inevitably creates complex and often biased deletion patterns. The off-target effects of a gRNA cocktail also accumulate in an additive manner. By contrast, CAMIO selects a single gRNA from each set and complexity can be added by increasing the number of sets. Thus, CAMIO confers every gRNA with some autonomy while achieving multiplexed mutagenesis as a whole. Off-target concerns in CAMIO can be adequately addressed by examining multiple independent mutations of similar kinds. Also, arrays of targeted mutations can be introduced into specific genetic backgrounds with CAMIO. For example, when performing CAMIO on the *chinmo* 5′ UTR, we purposely targeted a 2L chromosome arm that also carries transgenes for twin-spot MARCM (39). Hence, all the CAMIO *chinmo* indels were immediately ready for mosaic analysis.

In general, we recovered similar indel spectrums to what has been commonly described. For gRNAs that are inherently potent, like g1_4 for *chinmo* 5′ UTR, we obtained many indels around the cut site. Despite recovering numerous single-site deletions, we rarely see single-site deletions exceeding 30 bp in length. Therefore, the observed ease in creating diverse inter-target deletions by CAMIO is particularly valuable for systematic DNA dissection. In the case of CAMIO on *chinmo* 5′UTR, we recovered most of the predicted inter-target deletions with the exception of the ‘toxic’ g2_2 and g2_3. Notably, the largest inter-target deletion we have identified exceeds 1.5 kb in length. These observations suggest that we can be more aggressive in choosing more disperse gRNA targets to cover larger genomic regions.

After all, the capacity of CAMIO is mainly determined by how many gRNAs one can pack into a single set. Given the small size of gRNAs, we expect no problem in packing six to ten gRNAs without compromising the system. This intuition was largely supported by seeing reasonable recruitment frequencies for all six tandem gRNAs in the *chinmo* exon 2 set (Supplementary Figure S5). Nonetheless, there is still room for improvement on the gRNA selection process. For instance, increasing the distance between the U6 promoter and the gRNA set would likely make the selection more impartial. In sum, we have shown that the CAMIO system holds great promise for *in vivo* deletion analysis. Yet, our demonstrations have not nearly reached the limitations of CAMIO as far as the number of targets and size of DNA that can be evaluated in a single experiment.

We used CAMIO to perform deletion analysis on the 5′UTR of *chinmo*, which has important roles in governing Chinmo protein levels. There evidently exist multiple mechanisms governing *chinmo* expression throughout development. We successfully identified a 154 bp region responsible for Chinmo downregulation in the MB around pupa formation (Figures 3 and 4). The resulting elevated Chinmo expression affected MB morphogenesis (Figure 4), possibly due to abnormal neuronal fate transition. In addition, we found a large region (around g2_2 and g2_3) critical for embryo viability. While roles for Chinmo have been described in the brain (37,47,53), and Chinmo has also been identified as a downstream target of JAK/STAT in the testes (59), our data suggest an additonal essential role for Chinmo in embryonic development. The identification of discrete non-coding regions regulating different biological processes within a single UTR has exemplified the utility of CAMIO in resolving complex UTR functions. Given its multiplex and combinatorial power, CAMIO should also greatly aid the dissection of promoters, enhancers, long non-coding RNAs, DNA repeats and more.

CAMIO is intended for fine dissection of defined genomic regions rather than genome-wide analysis. For example, let us consider the classic *Drosophila* patterning gene *even-skipped* (*eve*). The *eve* locus (∼13 kb) contains multiple enhancers which control the seven stripes of *eve* expression. It took scientists years to uncover *eve's* sophisticated CREs by investing numerous transgenic synthetic reporters fused with genomic fragments upstream and downstream of the gene (60). If CAMIO were available, it would have offered an unique opportunity to scan for its large stripe identity enhancers (61) and further pinpoint small activator or repressor binding sites within stripe 2 enhancer (62) in their native sequence context.

In theory, CAMIO should work for any organism where a *bamP*-like promoter exists, and multiple progeny are produced. The *bam* gene and expression profile may be evolutionarily conserved. A putative murine ortholog, *Kiz (gm114)*, has been described as being enriched in undifferentiated spermatocytes and spermatids but absent or extremely low in undifferentiated spermatogonia (63). *Kiz* orthologs can be found in multiple species, including zebrafish, an ideal vertebrate candidate for CAMIO. We have pioneered CAMIO as a germline pipeline for arrayed CRISPR mutagenesis in *Drosophila*. Analogous systems, like CAMIO, may become a desirable genetic screening platform for interrogating the genome in multi-cellular organisms.

## Supplementary Material

gkaa177_Supplemental_File
