# Supplementary material for: CAMIO: a transgenic CRISPR pipeline to create diverse targeted genome deletions in Drosophila
Source: Nucleic Acids Res. 2020 Mar 18;48(8):4344–56. doi: 10.1093/nar/gkaa177 (PMC7192631; doi:10.1093/nar/gkaa177)
Supplement: gkaa177_Supplemental_File [file gkaa177_supplemental_file.pdf]

Supplementary Material

Supplementary Figure Legends

Supplementary Figure S1. Sequences of loss-of-ebony progeny from *bamP-Cas9*, *nosP-Cas9*, and *vasaP-Cas9* male founders. 30 *ebony* progeny were sequenced for each of the three conditions. 10 progeny were randomly selected from each male founder and three male founders were chosen for each germline Cas9. Their indels are arranged from small to large for comparison. Indel types are denoted by a "d" for deletion and an "i" for insertion, following by a number indicating the amount of loss or gain of nucleotides.

Supplementary Figure S2. Targeted mutagenesis of mCD8::GFP with pCis-{4gRNAs_mCD8}. pCis-{4gRNAs_mCD8} was created to target four different sites along the mCD8 coding sequence of UAS-mCD8::GFP. We analyzed 30 GFP-negative progeny from two different male founders. Each GFP-negative progeny carried a indel located at one of the four target sites. The location, size, and overall counts of these 30 indels are summarized here.

Supplementary Figure S3. Three *13XLexAop2-GFP* reporters for investigating *chinmo* 5' and 3' UTRs. Top: diagram of 13XLexAop2-GFP reporters flanked by both *chinmo* 5' and 3' UTRs, only the 5' UTR, or only the 3' UTR. Bottom: GFP transgene expression was induced and restricted in MB lineages by immortalizing a transient MB NB expression (*41A10-KD*) into a sustained MB NB production of these three GFPs, with this genetic setup: *DpnEE-KO-LexAp65; 13XLexAop2-GFP-UTRs; 41A10-KD*. Their expression profile is shown by immunostaining for GFP at two time points (48 and 72 hours after larval hatching, ALH). GFP with the 3' UTR is expressed at a much higher level than GFP with both 5' and 3' UTRs. Expression of GFP with the 5' UTR decreases over time. Green: GFP; Red: Deadpan. Scale bars: 20 μm.

Supplementary Figure S4. The small indels created by CAMIO in *chinmo* Exon 1, 2, and 3. We sequenced 984 progeny (300 each for individual exons, and additional 84 for Ex1-Ex2) from our CAMIO on *chinmo* 5' UTR experiment. The size, location, and corresponding stock ID of the predicted deletions (marked in red) and insertions (marked in blue) are depicted here.

Supplementary Figure S5. Examining gRNA array selection of *pCis-(6gRNAs_chinmo Exon2)*. We studied how frequently each gRNA gets selected after *nos-phiC31* mediated recombination by scoring progeny of male *nos-phiC31; pCis-(6gRNAs_chinmo Exon2)*. (A) gRNA choices of 92 progeny from 9 male founders are presented in this heat map. (B) Counts for each gRNA from all the founders are aggregated and whether there is bias in selection is tested by Chi-square test assuming equal distribution. g2_2 (6.5%, 6/92) and g2_3 (7.6%, 7/92) are underrepresented.

Supplementary Figure S6. Three 5'UTR-GFP-3'UTR reporters carrying small to large *chinmo* Exon 2 deletions for studying the region around g2_2 and g2_3 target sites. We generated three additional chinmo 5' UTR-GFP-3' UTR reporters: d (50 bps deletion around g2_3), bD (larger 158 bps deletion), and D23 (removing all the Exon 2 sequence upstream of g2_3). GFPs were induced in the same *41A10-KD* immortalization strategy. We did not observe difference in expression among the four GFP UTR reporters at three development time points: 48h AEL (after egg laying), 96h AEL and, white pupa. Scale bars: 30 μm.

Supplementary Figures

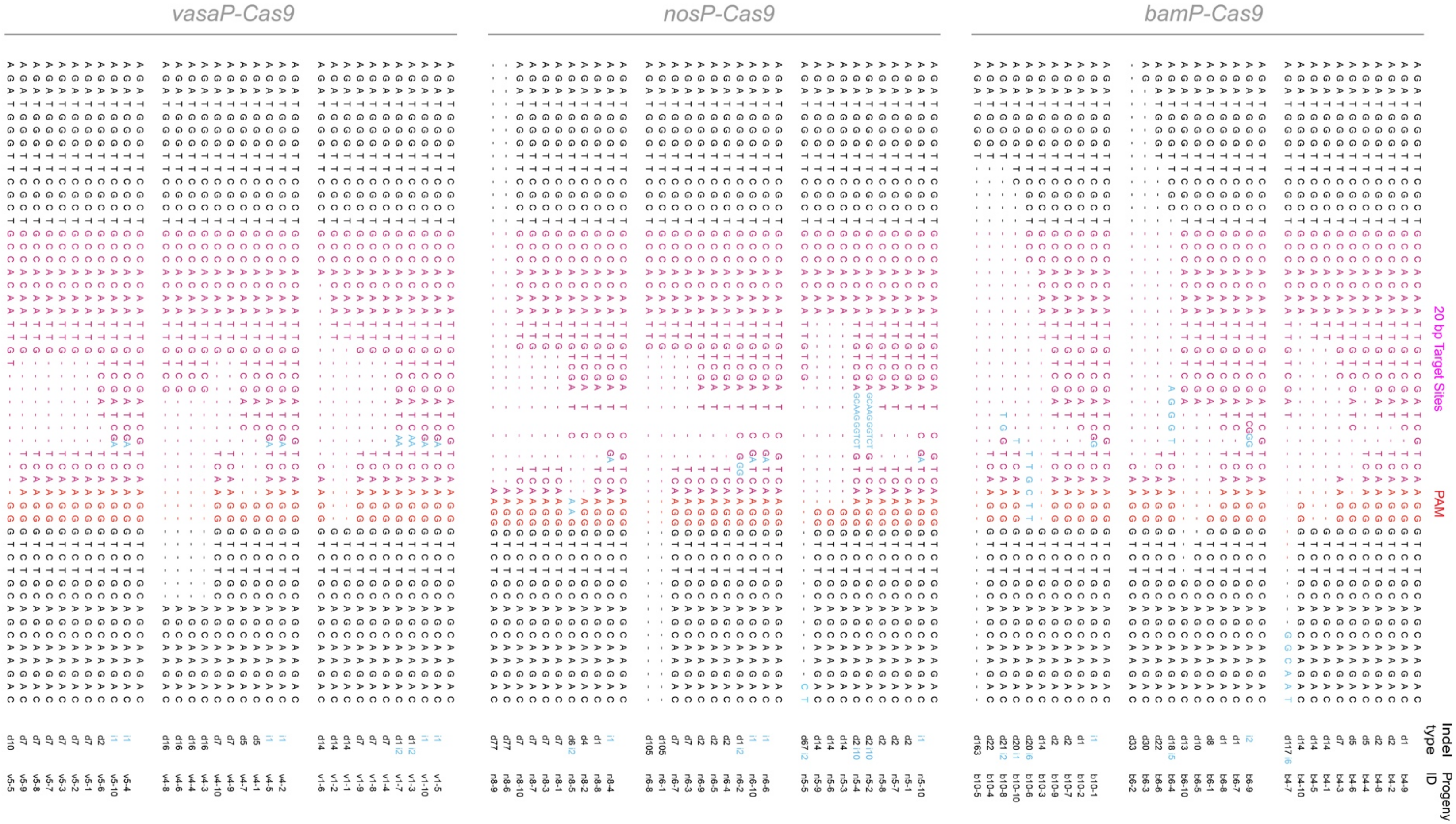

Supplementary Figure S1. Sequences of loss-of-ebony progeny from *bamP-Cas9*, *nosP-Cas9*, and *vasaP-Cas9* male founders. 30 *ebony* progeny were sequenced for each of the three conditions. 10 progeny were randomly selected from each male founder and three male founders were chosen for each germline Cas9. Their indels are arranged from small to large for comparison. Indel types are denoted by a “d” for deletion and an “i” for insertion, following by a number indicating the amount of loss or gain of nucleotide

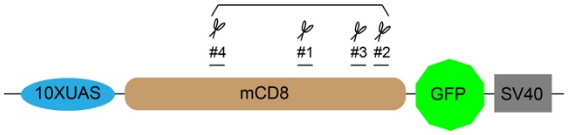

	#1	#2	#3	#4
Founder A	10	0	1	4
Founder B	9	2	1	3
Overall	62%	7%	7%	24%

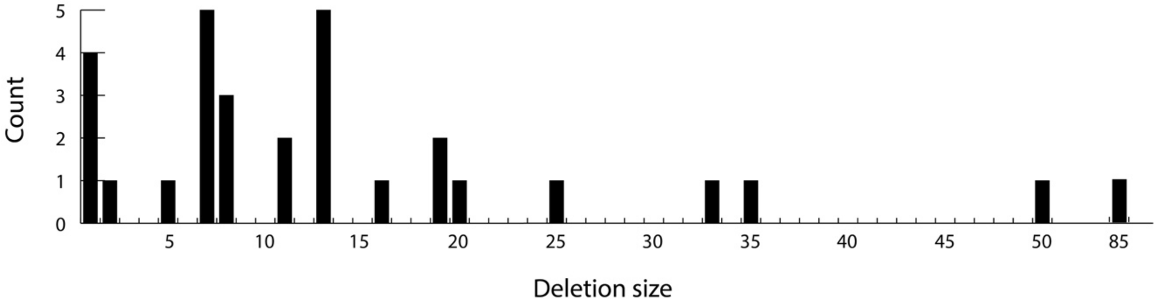

Supplementary Figure S2. Targeted mutagenesis of mCD8::GFP with pCis-{4gRNAs_mCD8}. pCis-{4gRNAs_mCD8} was created to target four different sites along the mCD8 coding sequence of UAS-mCD8::GFP. We analyzed 30 GFP-negative progeny from two different male founders. Each GFP-negative progeny carried a indel located at one of the four target sites. The location, size, and overall counts of these 30 indels are summarized here.

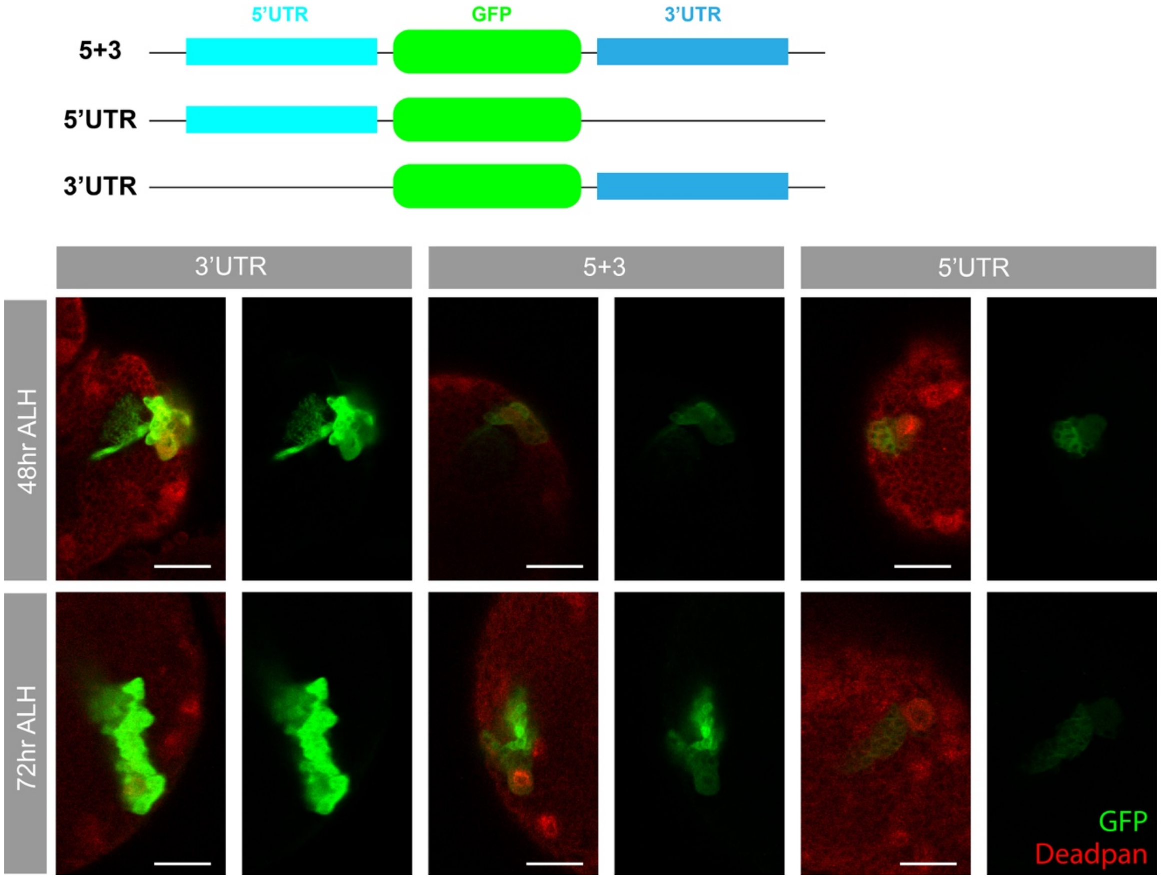

Supplementary Figure S3. Three *13XLexAop2-GFP* reporters for investigating *chinmo* 5' and 3' UTRs. Top: diagram of 13XLexAop2-GFP reporters flanked by both *chinmo* 5' and 3' UTRs, only the 5' UTR, or only the 3' UTR. Bottom: GFP transgene expression was induced and restricted in MB lineages by immortalizing a transient MB NB expression (*41A10-KD*) into a sustained MB NB production of these three GFPs, with this genetic setup: *DpnEE-KO-LexAp65; 13XLexAop2-GFP-UTRs; 41A10-KD*. Their expression profile is shown by immunostaining for GFP at two time points (48 and 72 hours after larval hatching, ALH). GFP with the 3' UTR is expressed at a much higher level than GFP with both 5' and 3' UTRs. Expression of GFP with the 5' UTR decreases over time. Green: GFP; Red: Deadpan. Scale bars: 20 μm.

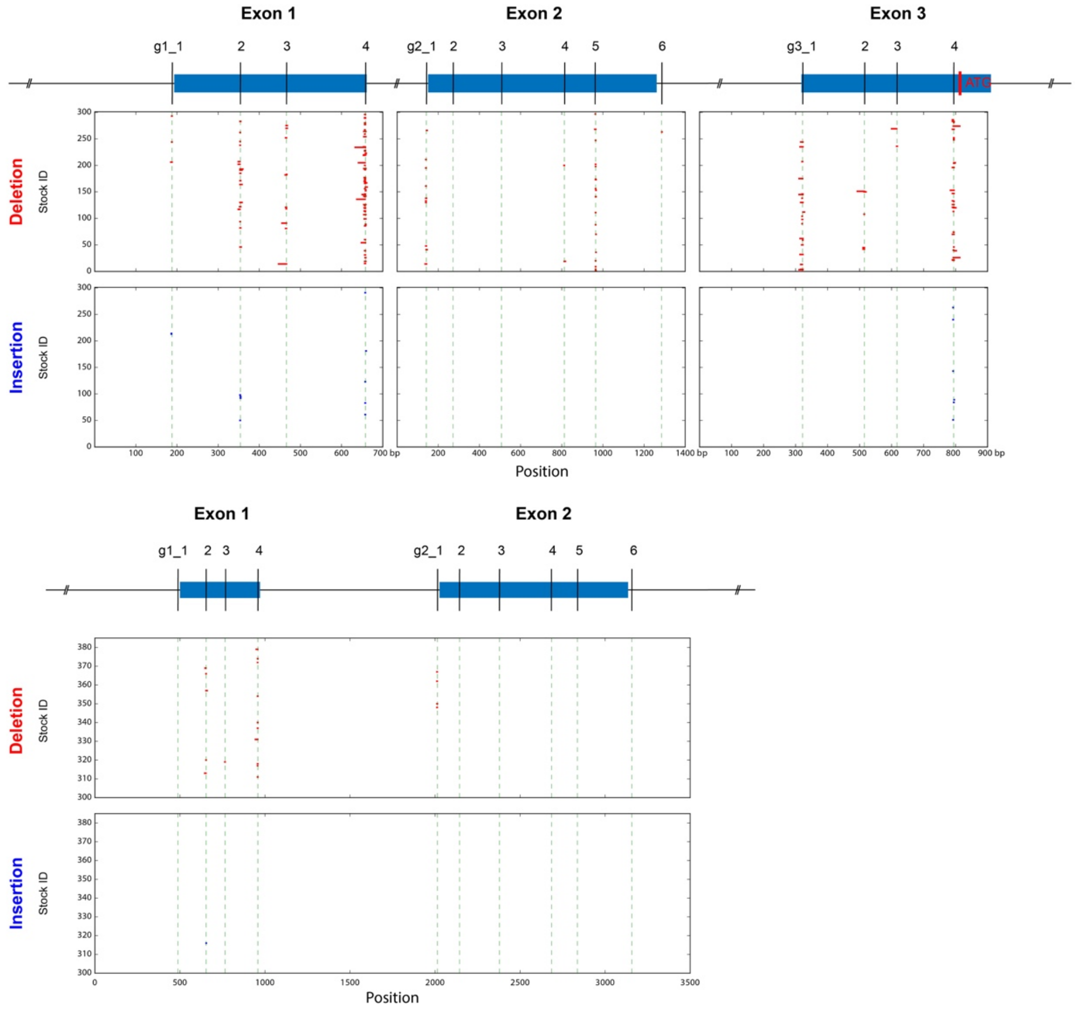

Supplementary Figure S4. The small indels created by CAMIO in *chinmo* Exon 1, 2, and 3. We sequenced 984 progeny (300 each for individual exons, and additional 84 for Ex1-Ex2) from our CAMIO on *chinmo* 5' UTR experiment. The size, location, and corresponding stock ID of the predicted deletions (marked in red) and insertions (marked in blue) are depicted here.

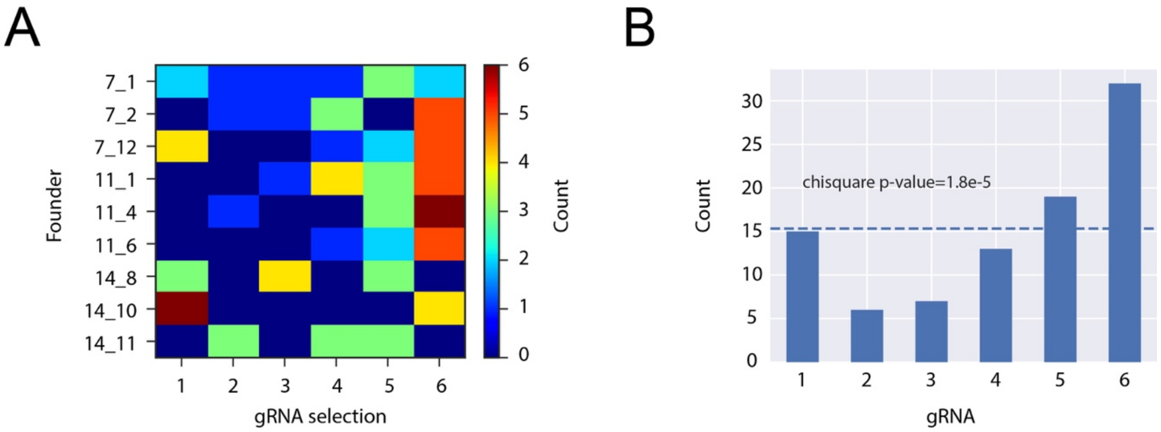

Supplementary Figure S5. Examining gRNA array selection of *pCis-(6gRNAs_chinmo Exon2)*. We studied how frequently each gRNA gets selected after nos-phiC31 mediated recombination by scoring progeny of male *nos-phiC31; pCis-(6gRNAs_chinmo Exon2*). (A) gRNA choices of 92 progeny from 9 male founders are presented in this heat map. (B) Counts for each gRNA from all the founders are aggregated and whether there is bias in selection is tested by Chi-square test assuming equal distribution. g2_2 (6.5%, 6/92) and g2_3 (7.6%, 7/92) are underrepresented.

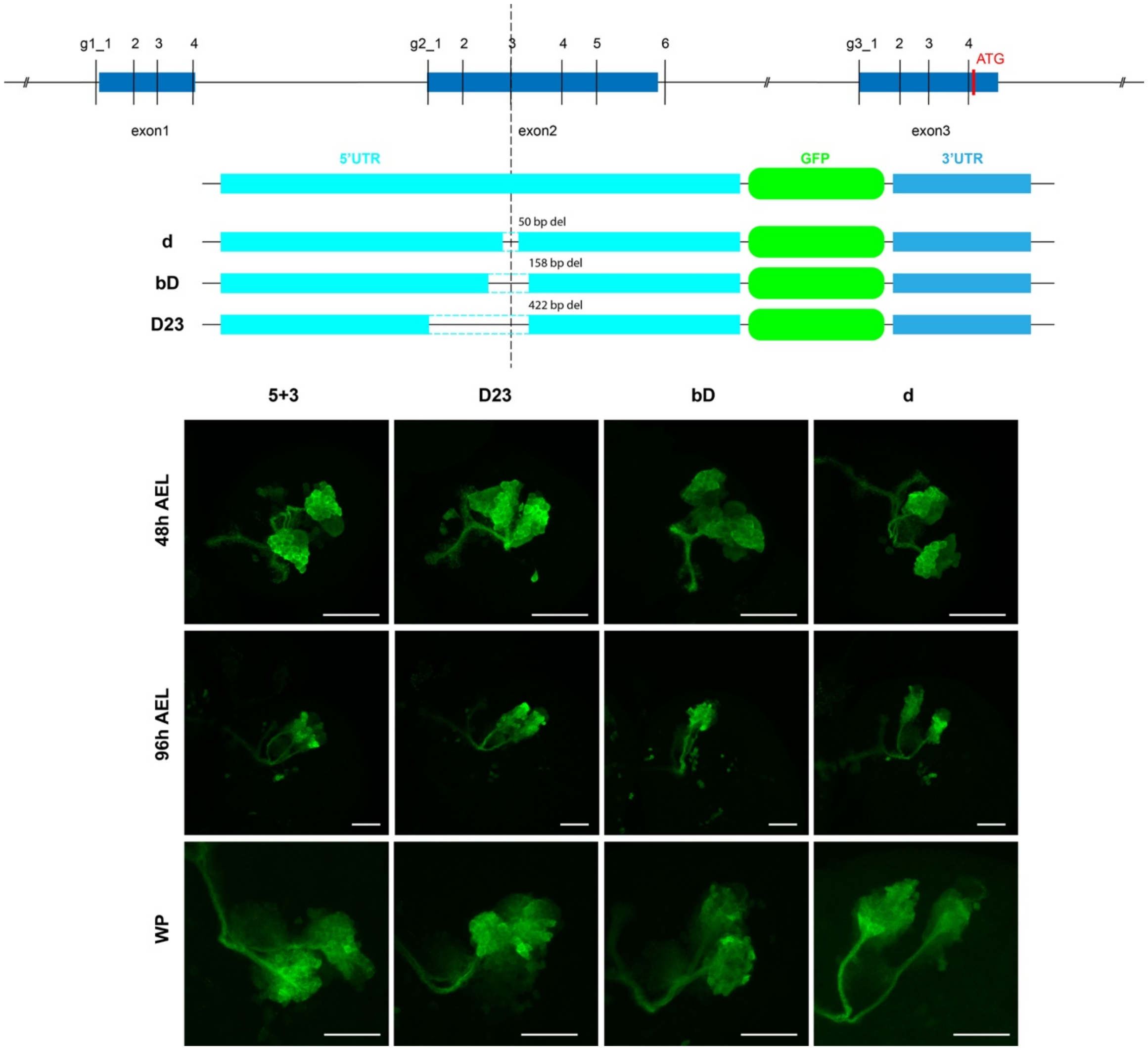

Supplementary Figure S6. Three 5'UTR-GFP-3'UTR reporters carrying small to large *chinmo* Exon 2 deletions for studying the region around g2_2 and g2_3 target sites. We generated three additional chinmo 5' UTR-GFP-3' UTR reporters: d (50 bps deletion around g2_3), bD (larger 158 bps deletion), and D23 (removing all the Exon 2 sequence upstream of g2_3). GFPs were induced in the same *41A10-KD* immortalization strategy. We did not observe difference in expression among the four GFP UTR reporters at three development time points: 48h AEL (after egg laying), 96h AEL and, white pupa. Scale bars: 30 μm.
